# Tumor-associated macrophages related signature in glioma

**DOI:** 10.18632/aging.203968

**Published:** 2022-03-24

**Authors:** Lin-Jian Wang, Yimeng Xue, Yongli Lou

**Affiliations:** 1 Advanced Medical Research Center of Zhengzhou University, Zhengzhou Central Hospital Affiliated to Zhengzhou University, Zhengzhou 450007, China; 2 Savaid Medical School, University of Chinese Academy of Sciences, Beijing 100049, China; 3 Department of Neurosurgery, Zhengzhou Central Hospital Affiliated to Zhengzhou University, Zhengzhou 450007, China

**Keywords:** glioma, tumor-associated macrophages, tumor microenvironment, prediction model, risk score

## Abstract

Background: Glioma is the most common malignant primary tumor with a poor prognosis. Infiltration of tumor-associated macrophages (TAMs) is a hallmark of glioma. However, the regulatory mechanism of TAMs and the prognostic value of related signature in glioma remain unclear.

Methods: TAMs were analyzed by EPIC, MCPCOUNTER and XCELL methods in multiple cohorts, including the TCGA merged GBMLGG, CGGA mRNAseq-325, and CGGA mRNAseq-693. Weighted correlation network analysis (WGCNA) were performed to identify candidate hub genes that might be related to TAMs. The prognostic genes were selected by Univariate Cox regression, Kaplan-Meier analysis and the least absolute shrinkage and selection operator (LASSO) multivariate Cox regression algorithm, and were used to construct a high efficacy prediction model.

Results: Compared with LGG, TAMs of GBM in the TCGA merged GBMLGG, CGGA mRNAseq-693, and CGGA mRNAseq-325 cohorts were increased, and high TAMs levels predicted poorer overall survival for gliomas. The prediction model constructed by nine prognostic genes was highly efficient. The TAMs related risk-score was an independent risk factor for glioma. Moreover, high risk score was correlated with an increased population of TAMs in glioma, as well as the high immune scores, stromal scores and ESTIMATE scores.

Conclusions: Increased TAMs might be an immune evasion mechanism of glioma. In addition, our findings suggested that TAMs-related signature was a valuable prognostic biomarker in glioma and provided therapeutic targets for glioma.

## INTRODUCTION

Glioma is the most common malignant brain tumor in adults [1]. It is generally difficult to treat due to extensive proliferation, invasion, angiogenesis, immunosuppression, and resistance to conventional treatments [2, 3]. Compared with low-grade glioma (LGG, grade II and III), glioblastoma (GBM, WHO grade IV) is more lethal [4]. Even with large surgical resection followed by combined radiotherapy and temozolomide chemotherapy, the median survival of glioblastoma is only 16 months [5].

In solid tumors, a variety of non-cancer cells, including various immune cells, inflammatory cells, vascular cells, fibrotic cells, and even adipocytes, together with cancer cells constitute the tumor microenvironment (TME) [6]. The main tumor-infiltrating immune cells in the tumor microenvironment of glioma are tumor-associated macrophages (TAMs), including blood-circulating monocytes and tissue resident microglia, accounting for about 30-50% of the infiltrating immune cells in GBM [7]. TAMs promote the progression and metastasis of cancer in a variety of ways, such as releasing cancer-promoting growth factors and cytokines, enhancing tumor invasion, inhibiting immune cell function, and stimulating angiogenesis [8–10]. Similar findings have been noticed in gliomas, and TAMs have also been found to mediate drug resistance in glioma immunotherapy [11]. Exploring the regulatory mechanism of TAMs and determining the prognostic value of TAMs related signature will be promising for improvement of the treatment of gliomas.

In this study, we analyzed the tumor-associated macrophages in the glioma by EPIC, MCPCOUNTER and XCELL methods, and found that compared with LGG, TAMs were increased in GBM in the TCGA dataset. The differentially expressed genes (DEGs) between LGG and GBM were selected for weighted correlation network analysis (WGCNA) to identify candidate modules and hub genes that might regulate TAM in glioma. Subsequently, the hub genes were analyzed by univariate cox regression and Kaplan-Meier. The hub genes significantly related to overall survival (OS) were extracted to perform the least absolute shrinkage and selection operator (LASSO) multivariate cox regression algorithm. Finally, nine positive prognostic genes (MYL12A, MSN, S100A4, CHI3L1, PLAUR, EMP3, CASP4, TIMP1 and CCDC109B) were screened out and used to construct a high efficacy prediction model. Moreover, through immune landscape analysis, we found that the risk score was significantly related to tumor microenvironment. In conclusion, we revealed the relationship between TAM and malignancy of glioma, demonstrated the value of TAM related signature in predicting the prognosis of glioma, and provided potential targeted therapy for glioma.

## MATERIALS AND METHODS

### Datasets and samples

The expression data and clinical data of merged GBMLGG dataset from The Cancer Genome Atlas (TCGA) were downloaded from University of California Santa Cruz (UCSC) Xena browser (https://xenabrowser.net/datapages/) [12]. The samples with missing data on survival and WHO grade were excluded in this study, and 674 glioma patients we finally obtained in the TCGA dataset. The RNA-seq data and clinical data of mRNAseq-693 and mRNAseq-325 cohorts were downloaded from the Chinese Glioma Genome Atlas (CGGA) data portal (http://www.cgga.org.cn/) [13] Finally, 656 and 309 glioma patients were enrolled in this study, respectively. In addition, three LGG and three GBM samples were collected from patients undergoing surgical treatment from November 2019 to December 2020 in Zhengzhou Central Hospital Affiliated to Zhengzhou University. The clinical diagnosis was confirmed by immunohistochemical staining in the pathology department This study was approved by the institutional review board of Zhengzhou Central Hospital Affiliated to Zhengzhou University, and informed consents were obtained from all patients.

### Immune microenvironment analysis

The abundance of tumor-infiltrating macrophage cells in glioma were evaluated by using EPIC, MCPCOUNTER and XCELL algorithms on the TIMER2 platform (http://timer.cistrome.org/) [14]. The ESTIMATE scores, Immune scores and Stromal scores of gliomas were calculated using the R package “estimate”.

### Identification of DEGs and GO enrichment analysis

The differently expressed genes (DEGs) (adjusted p-value < 0.05 and |log_2_FC| ≥ 1) were identified in the TCGA dataset by using the R package“ limma” [15]. Gene enrichment analysis was conducted using the KOBAS-i (http://bioinfo.org/kobas) [16].

### Construction of the risk score model

DEGs and clinical traits were incorporated to perform Weighted correlation network analysis (WGCNA) using R package “WGCNA”, the tumor-infiltrating macrophage related hub genes were identified. Univariate Cox regression analysis was performed to identify the hub genes which significantly related to overall survival (OS). After that, the least absolute shrinkage and selection operator (LASSO) multivariate Cox regression algorithm was performed using the R package “glmnet”. to reduce the number of predictors and screen for significant predictors [17, 18]. Finally, the positive genes were screened out, and the coefficients in the risk score signature were constructed based on the most suitable penalty parameter λ. The risk score formula was as follow:


$$Risk\;score=\sum_{i=1}^{n}\left(Coef_{i}*Exp_{i}\right)$$

where Coef_i_ is the coefficient, and Exp_i_ is the normalized expression of each signature gene.

### Immunohistochemical staining

The tissue sections were incubated with anti-CHI3L1 (abcam, ab255297, 1:250), anti-MSN (abcam, ab151542, 1:250) or anti-TIMP1 (abcam, ab211926, 1:250) primary antibody overnight at 4° C. After washing three times, the sections were incubated with horseradish peroxidase-conjugated goat anti-rabbit IgG secondary antibody for 20 minutes, followed by staining with diaminobenzidine. Finally, the sections were counterstained with hematoxylin.

### Statistical analysis

One-way ANOVA, Wilcoxon test and t test were used to analyze the significance of differences in gene expression levels and macrophage infiltration levels. Univariate, multivariate, LASSO cox regression and Kaplan-Meier analyses were performed to screen positive prognostic genes and evaluate the risk signature using the R packages “glmnet” and “survival”. Roc curve was drawn using the R package “survivalROC.” All statistical analyses were performed using GraphPad Prism 8, R software and SPSS, and a p value of less than 0.05 was considered statistically significant.

### Ethics statement

The studies involving human participants were reviewed and approved by the medical ethics committee of the Zhengzhou Central Hospital Affiliated to Zhengzhou University. Informed consents were obtained from all individual participants included in the study.

## RESULTS

### Analysis of tumor-associated macrophages (TAMs) in glioma

**Figure 1:**
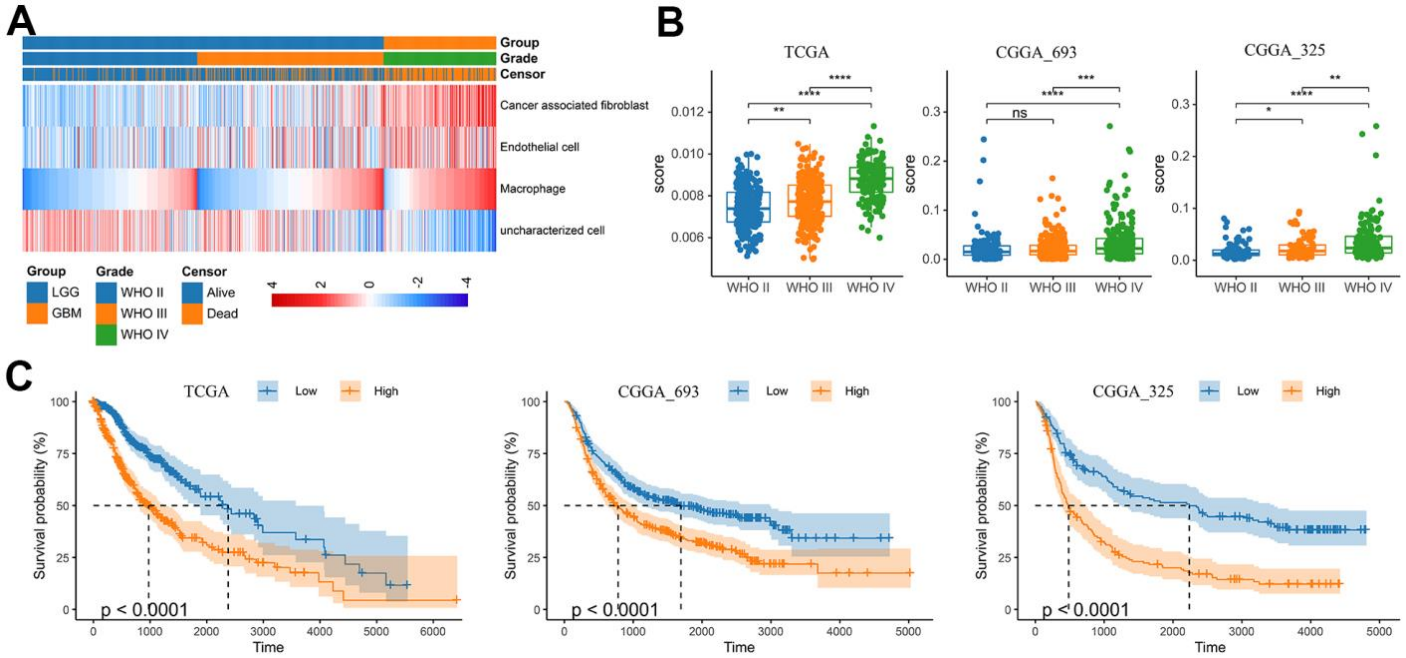
**Analysis of tumor infiltrating macrophages (TAM) in glioma.** (**A**) Heatmap was drawn to depict the TAM in glioma from the TCGA merged GBMLGG cohort. (**B**) TAMs were increased in GBM in the TCGA merged GBMLGG, CGGA mRNAseq-693 and CGGA mRNAseq-325 cohorts, respectively. (**C**) Kaplan-Meier overall survival curves displayed that increased TAM was related to the poor prognosis and lower survival rate of glioma. *, P< 0.05; **, P< 0.01; ***, P< 0.001, ****, P< 0.0001.

With the progress of glioma, tumor cells secrete a large number of chemokines to recruit immune cells. The increase in the proportion of macrophages in gliomas is related to the degree of malignancy [19]. Previous study had assessed the accuracy of 7 tools at estimating different immune cells from bulk RNA-seq data by developing a systematic approach for benchmarking such computational methods. Finally, the EPIC, MCPCOUNTER and XCELL methods were recommend to predict the level of tumor infiltrating macrophages [20]. Therefore, we analyzed the RNA-seq data from glioma patients in the CGGA mRNAseq-325, CGGA mRNAseq-693 and TCGA merged GBMLGG cohorts by these three methods to characterize the TAM in gliomas (Figure 1 and Supplementary Figure 1). The results showed that compared with LGG, the level of macrophages in GBM was significantly increased (Figure 1A, 1B and Supplementary Figure 1). In addition, the level of macrophages was significantly correlated with overall survival of gliomas (Figure 1C). In different WHO grade, age, MGMT status and IDH mutation status group, TAMs also showed significant differences. However, there was no difference in gender group (Supplementary Figure 2).

### Identification of differentially expressed genes (DEGs) related to TAM

**Figure 2:**
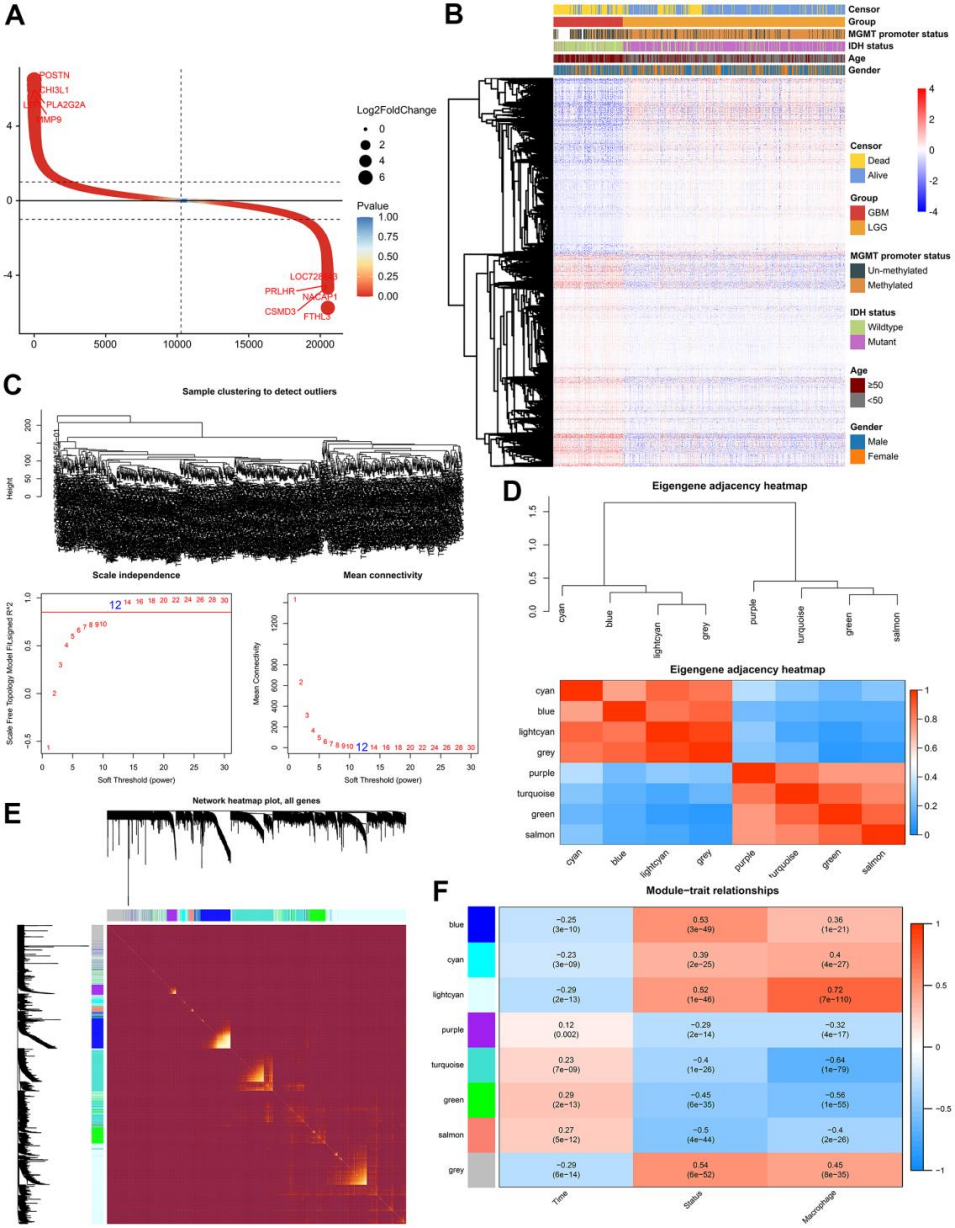
**Weighted correlation network analysis.** (**A**) Scatter plot showed 2116 up-regulated genes and 1752 down-regulated genes. (**B**) Cluster dendrogram demonstrating good separation between LGG and GBM. (**C**) Sample Dendrogram and soft-thresholding powers. (**D**) Clustering tree and adjacency heatmap of modules. (**E**) TOM diagram of the relationship between gene clusters and modules in each module of WGCNA. (**F**) Module-trait relationships indicated the light-cyan module was most related to the level of TAM in glioma.

The differentially expressed genes (DEGs) between LGG and GBM were identified using R package “limma”. In the TCGA GBMLGG cohort, 3868 DEGs (adjusted p-value < 0.05 and |log_2_FC| ≥ 1) were screened out, including 2116 up-regulated genes and 1752 down-regulated genes, respectively (Figure 2A, 2B). Thereafter, we performed Weighted Correlation Network Analysis (WGCNA) to determine the co-expression modules associated with macrophage infiltration (Figure 2). A total of 8 modules were identified from the co-expression network (Figure 2D, 2E), among which the light-cyan module was most related to TAM (Figure 2F).

**Figure 3:**
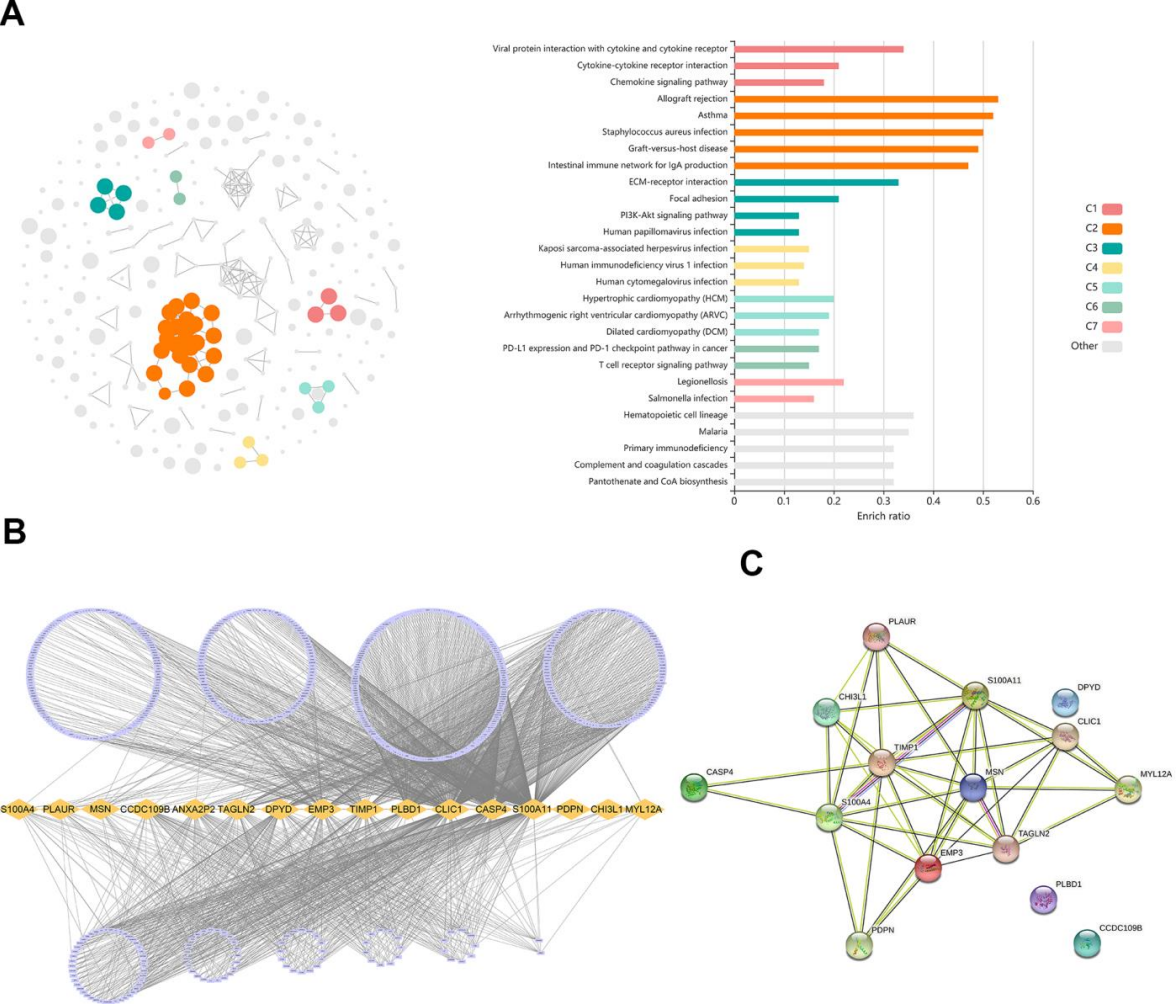
**Identification of differentially expressed genes (DEGs) related to TAM.** (**A**) GO analysis showed the DEGs were significantly enriched in Immunology signaling pathways. (**B**) 16 hub genes were identified in the light-cyan module. (**C**) The function and interaction of hub genes.

1372 genes were identified in the light-cyan module. GO enrichment analysis showed that these genes were significantly enriched in immunology signaling pathways such as the cytokine-cytokine receptor pathway, chemokine signaling pathway, PD-L1 expression and PD-1 checkpoint pathway in cancer (Figure 3A). In addition, 16 hub genes (S100A4, PLAUR, MSN, CCDC109B, ANXA2P2, TAGLN2, DPYD, EMP3, TIMP1, PLBD1, CLIC1, CASP4, S100A11, PDPN, CHI3L1 and MYL12A) were identified from the light-cyan module through WGCNA analysis (Figure 3B, 3C).

### Construction of the risk score signature

**Figure 4:**
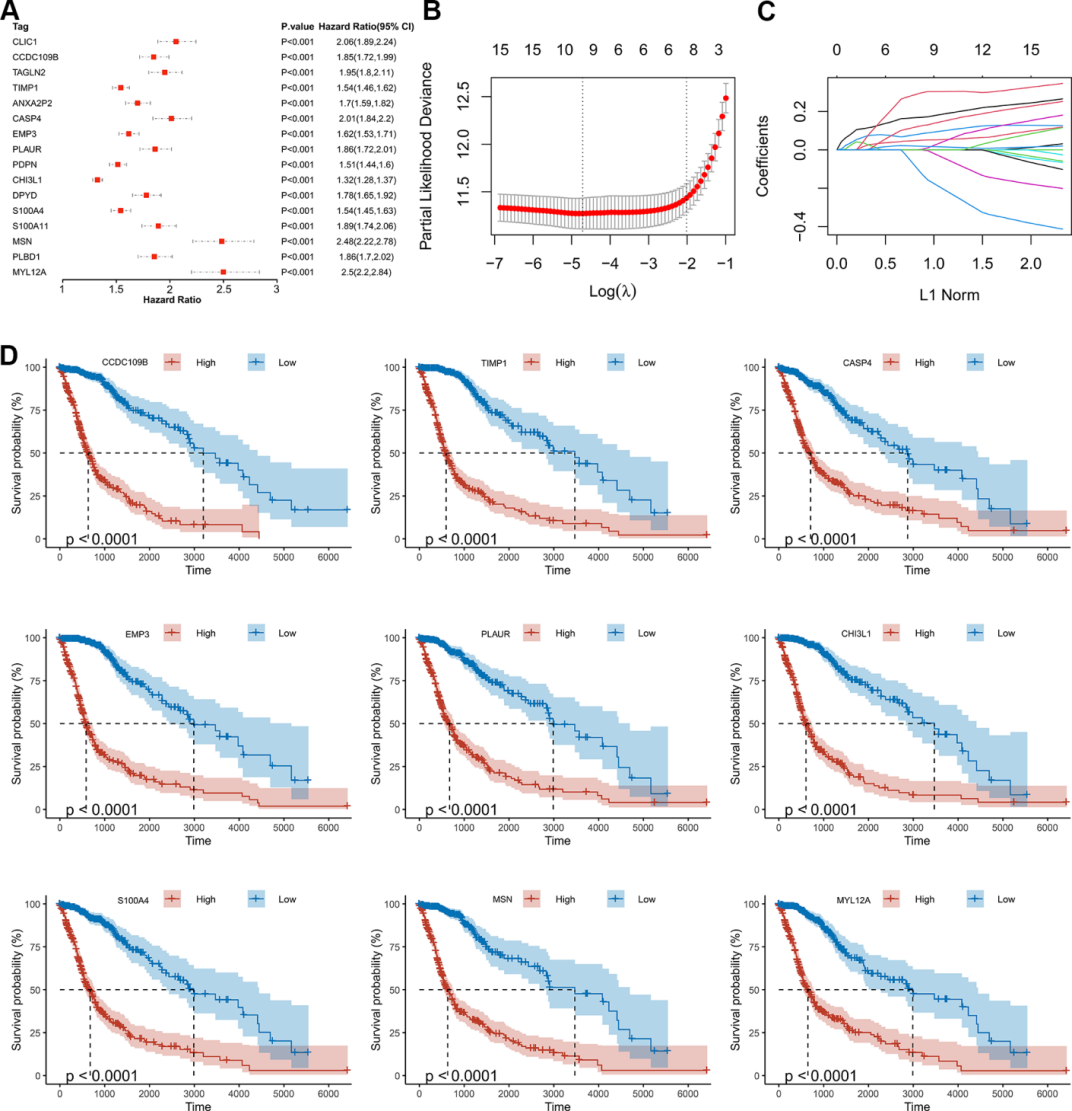
**Screening of the prognostic genes in the TCGA dataset.** (**A**) Univariate Cox regression analysis of 16 hub genes in the TCGA dataset. (**B**) Partial likelihood deviance of different numbers of variables revealed by the LASSO regression model. (**C**) LASSO coefficient profiles of the selected hub genes. (**D**) Kaplan-Meier curves displayed highly expressed genes screened out by LASSO were significantly related to poor prognosis in the TCGA dataset.

**Figure 5:**
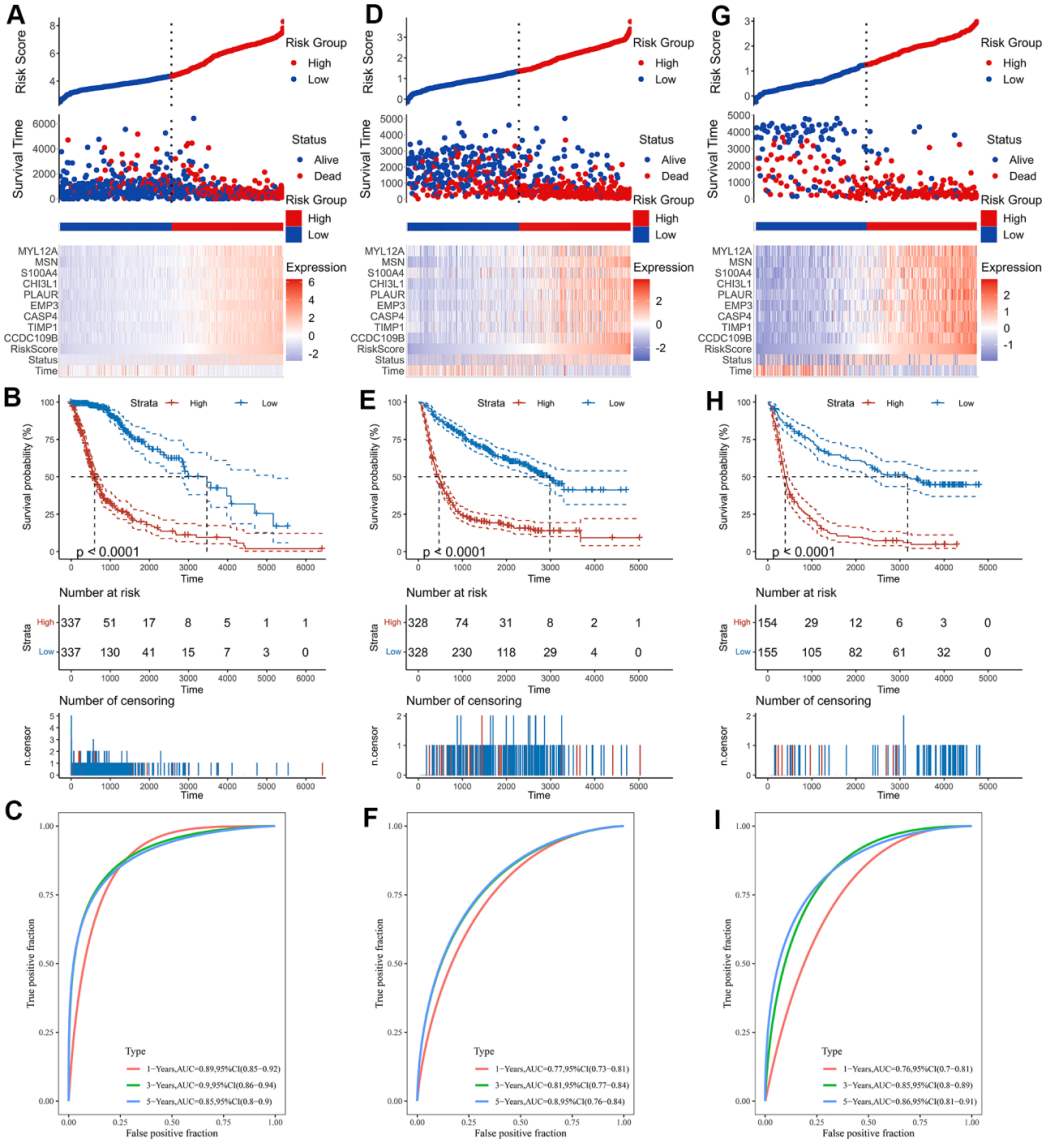
**Construction of the risk score signature.** (**A**) Risk signature in the TCGA cohort. (**B**) Kaplan-Meier analysis according to risk score in the TCGA cohort. (**C**) The ROC curves depicting the sensitivity and specificity of risk score in predicting the 1-, 3- and 5-year survival in the TCGA cohort. (**D**) Risk signature in the CGGA mRNAseq-693 cohort. (**E**) Kaplan-Meier analysis according to risk score in the CGGA mRNAseq-693 cohort. (**F**) The ROC curves depicting the sensitivity and specificity of risk score in predicting the 1-, 3- and 5-year survival in the CGGA mRNAseq-693 cohort. (**G**) Risk signature in the CGGA mRNAseq-325 cohort. (**H**) Kaplan-Meier analysis according to risk score in the CGGA mRNAseq-325 cohort. (**I**) The ROC curves depicting the sensitivity and specificity of risk score in predicting the 1-, 3- and 5-year survival in the CGGA mRNAseq-325 cohort.

As shown by the Univariate Cox regression analysis, all the 16 hub genes were significantly associated with prognosis in the TCGA dataset (Figure 4A). Subsequently, we performed the least absolute shrinkage and selection operator (LASSO) Cox regression algorithm to analyze the 16 hub genes in the TCGA dataset. Nine prognostic-related genes (MYL12A, MSN, S100A4, CHI3L1, PLAUR, EMP3, CASP4, TIMP1 and CCDC109B) were screened out based on the minimum criteria in the TCGA dataset (Figure 4B, 4C). The results of Kaplan-Meier analyses displayed that the expression of the nine prognostic-related genes was significantly correlated with the overall survival of glioma patients in the TCGA dataset (Figure 4D). Therefore, these nine genes were finally selected to construct the risk score signature (Figure 5A, 5D, 5G). Kaplan-Meier survival analyses showed that high risk score was related to the poor prognosis and lower survival rate of glioma both in the CGGA mRNAseq-325, CGGA mRNAseq-693 and TCGA merged GBMLGG cohorts (Figure 5B, 5E, 5H). In order to assess the sensitivity and specificity of risk score in predicting the 1-, 3- and 5-year survival of glioma patients, we conducted ROC curve analyses in three cohorts and found that the predictive accuracy of the risk score was very high (Figure 5C, 5F, 5I).

### The relationship between risk score and TAM

**Figure 6:**
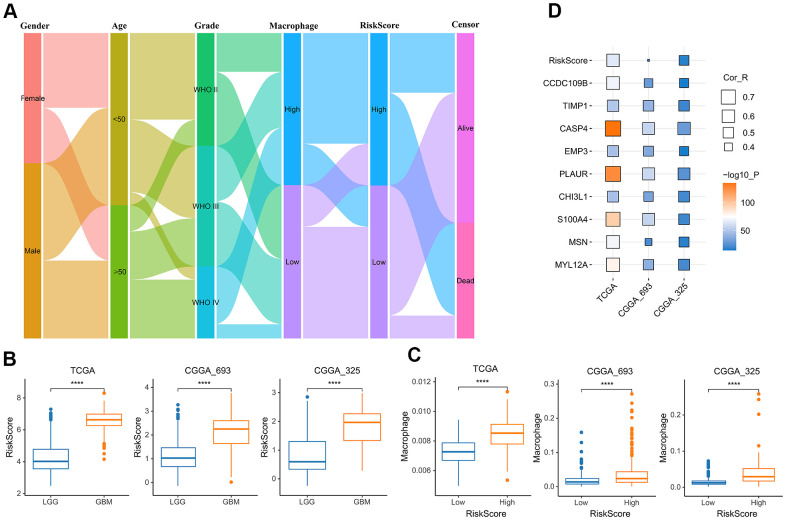
**The relationship between risk score and TAM.** (**A**) Sankey Diagram displayed the distribution of the survival status, age, WHO grade, risk score and TAM of glioma patients in the merged GBMLGG cohort. (**B**) Boxplot showed the risk scores of GBM were higher than those of LGG in the cohort of TCGA merged GBMLGG, CGGA mRNAseq-693 and CGGA mRNAseq-325, respectively. (**C**) Boxplot showed the TAM of high-risk group was higher than that of low-risk group in the cohort of TCGA merged GBMLGG, CGGA mRNAseq-693 and CGGA mRNAseq-325, respectively. (**D**) Correlation analysis showed that the risk score and nine prognostic genes were significantly related to TAM in the cohort of TCGA merged GBMLGG, CGGA mRNAseq-693 and CGGA mRNAseq-325, respectively. *, P< 0.05; **, P< 0.01; ***, P< 0.001, ****, P< 0.0001.

To better understand the relationship between risk score and TAM, we analyzed the distribution of the survival status, age, WHO grade, risk score and TAM of glioma patients (Figure 6A). The risk scores of gliomas in GBM were significantly higher than the corresponding LGG subtype (Figure 6B). In addition, the infiltration level of macrophages was increased in the high-risk group (Figure 6C). The risk score also was closely related to age, IDH mutation status and MGMT status (Supplementary Figure 3). In addition to the risk score, all nine genes that constructed the risk signature were significantly associated with the TAM in glioma (Figure 6D).

### Immune microenvironment analysis of glioma

**Figure 7:**
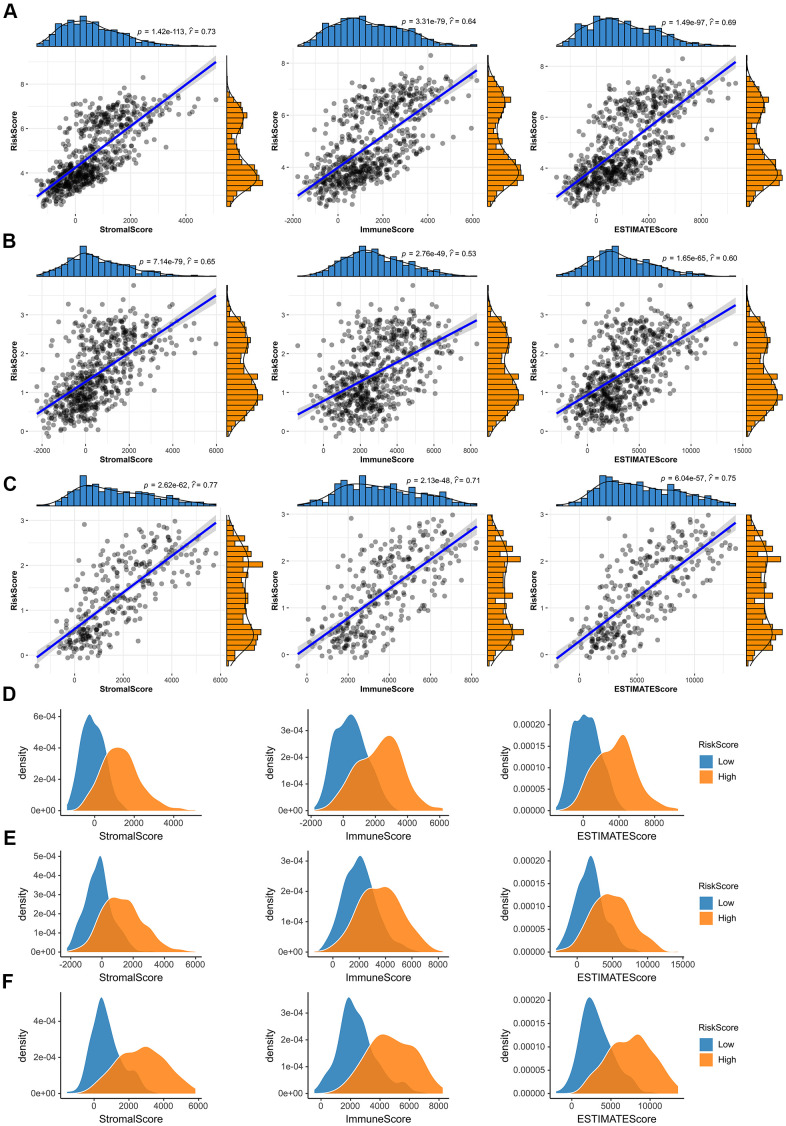
**Immune microenvironment analysis of glioma.** (**A**–**C**) In the TCGA merged GBMLGG, CGGA mRNAseq-693 and CGGA mRNAseq-325 cohorts, the risk score was significantly correlated with the immune scores, stromal scores and ESTIMATE scores, respectively. (**D**–**F**) Compared with low-risk group, the immune scores, stromal scores and ESTIMATE scores of high-risk group were higher in the TCGA merged GBMLGG, CGGA mRNAseq-693 and CGGA mRNAseq-325 cohorts.

After that, we investigated whether the risk score was associated with tumor immune microenvironment in the TCGA dataset (Figure 7). The results of correlation analysis showed that risk scores were positively correlated with immune scores, stromal scores and ESTIMATE scores both in the CGGA mRNAseq-325, CGGA mRNAseq-693 and TCGA merged GBMLGG cohorts (Figure 7A–7C). And the immune scores, stromal scores and ESTIMATE scores of the high-risk group were higher than those of the low-risk group (Figure 7D–7F).

### TAM related signature is an independent risk factor for glioma

**Figure 8:**
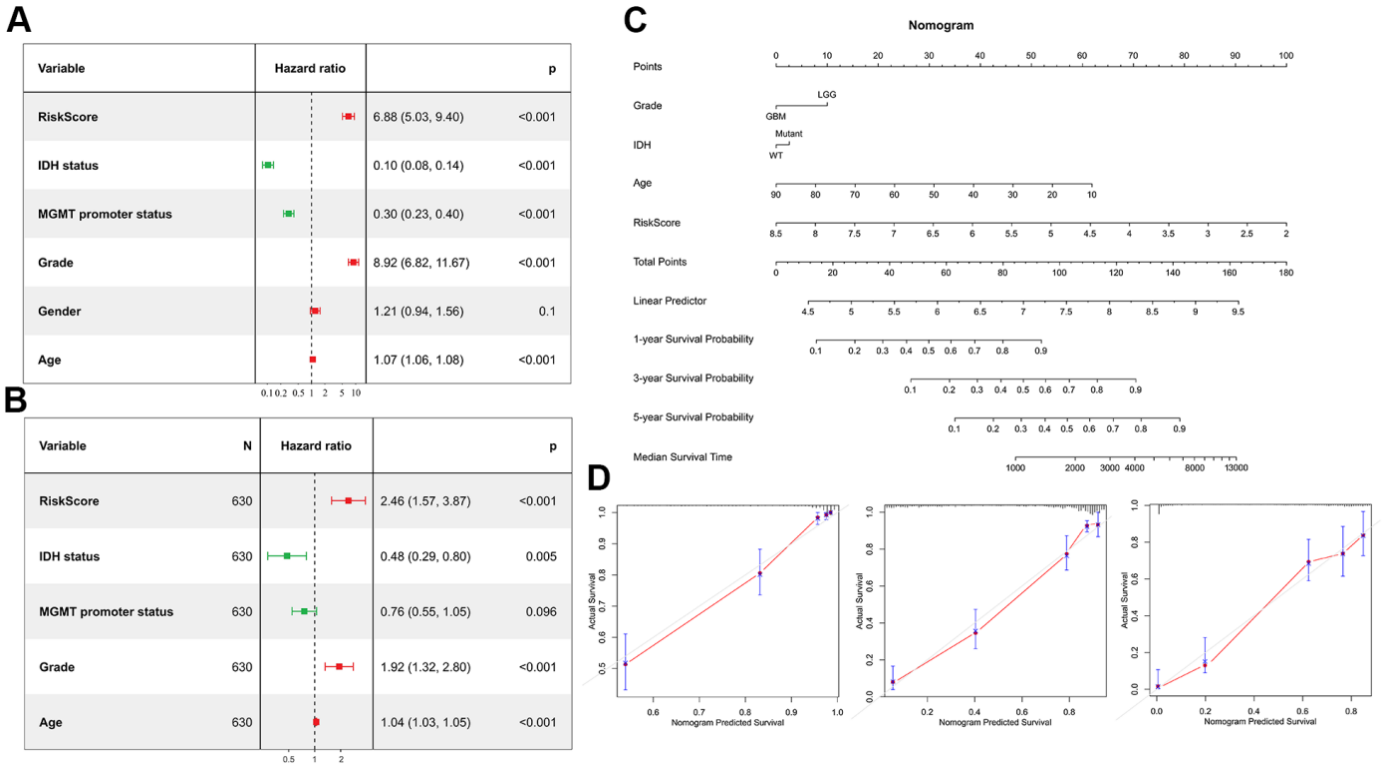
**Risk score is an independent prognostic factor.** (**A**) Univariate Cox regression analyses showed the clinical features such as the risk score, age, MGMT promoter status, WHO grade and IDH status were significantly correlated with prognosis. (**B**) Multivariate Cox analysis showed the risk score remained associated with the prognosis. (**C**) Nomogram was used to predict prognosis in patients at 1-, 3-, and 5-years in the CGGA dataset. (**D**) Calibration curve for the nomogram predicting 1-, 3-, and 5-years overall survival.

Univariate Cox regression analysis was performed to investigate whether the risk score was an independent prognostic factor. As shown in the Figure 8A, the risk score, age, MGMT promoter status, WHO grade and IDH status were significantly correlated with prognosis. Multivariate Cox regression analysis also revealed that the risk score, IDH status, age and WHO grade were significantly correlated with prognosis (Figure 8B), indicating that the risk score was an independent prognostic factor for glioma. We then built a survival nomogram prediction model based on independent prognostic parameters for the OS of glioma patients (Figure 8C). Finally, the calibration curves were drawn, and the results displayed excellent agreement between observation and prediction both in the CGGA mRNAseq-325, CGGA mRNAseq-693 and TCGA merged GBMLGG cohorts (Figure 8D).

### Validation the expression of the prognostic genes

**Figure 9:**
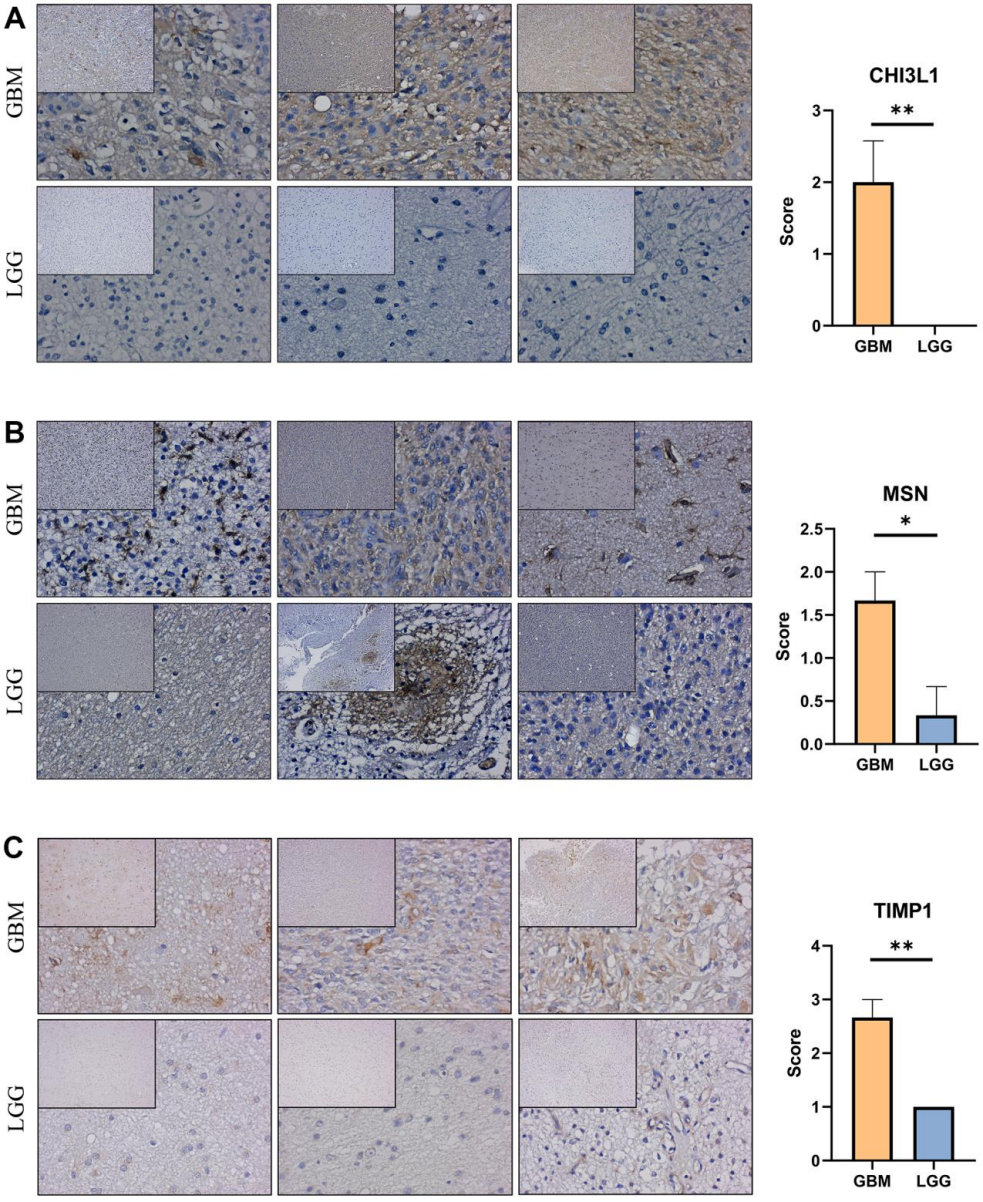
**Expression verification of the prognostic genes.** (**A**–**C**) Immunohistochemical staining analysis of the protein levels of CHI3L1, MSN and TIMP1 between the low-grade and high-grade gliomas. *, P< 0.05; **, P< 0.01; ***, P< 0.001.

Immunohistochemical staining were performed to validate the expression of the genes that constructed the risk signature in glioma. Similar to the expression pattern of CGGA mRNAseq-325, CGGA mRNAseq-693 and TCGA merged GBMLGG cohorts, CHI3L1, MSN and TIMP1 were expressed higher in high-grade gliomas than in low-grade gliomas (Figure 9).

### TIMP1 affects migration and proliferation of glioma cells

**Figure 10:**
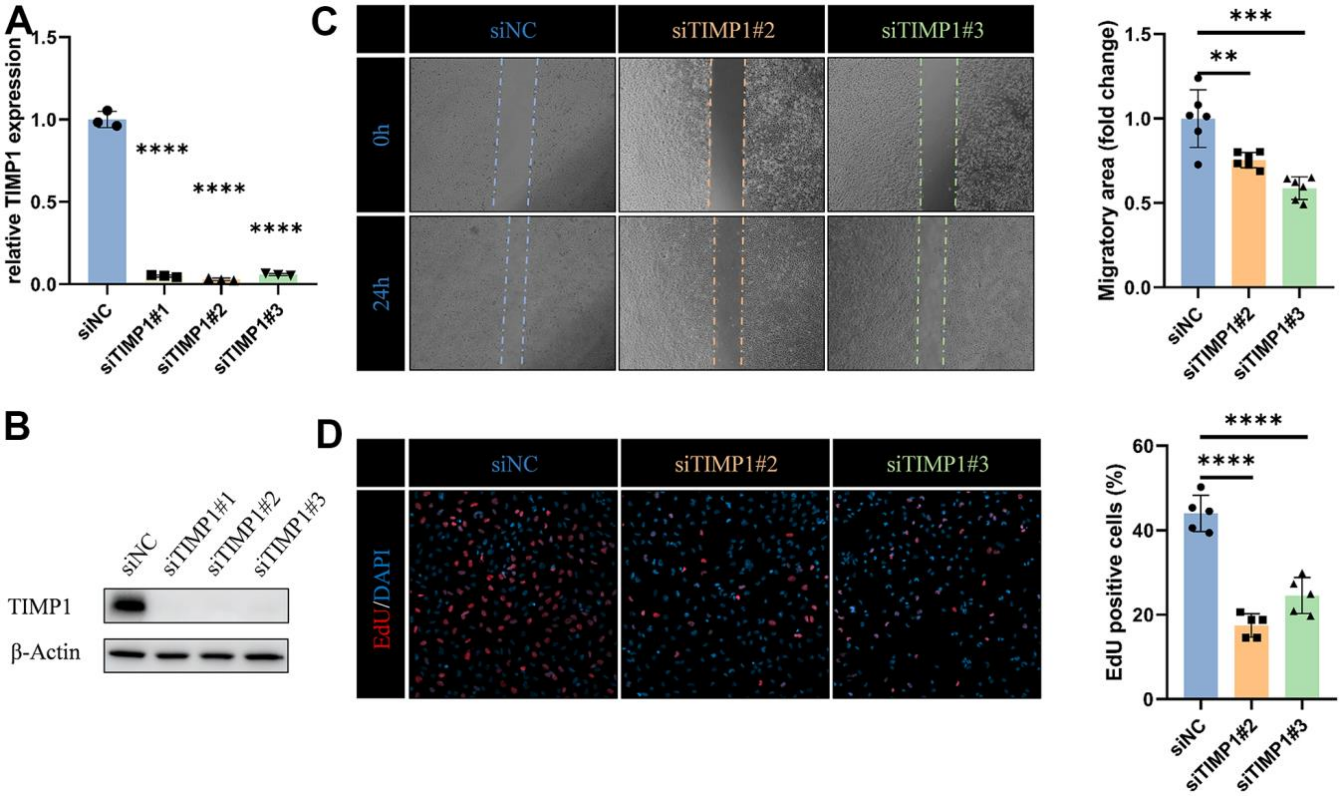
**TIMP1 affects migration and proliferation of glioma cells.** (**A**) qRT-PCR and (**B**) western blot analysis of TIMP1 knockdown efficiency in LN229 cells. (**C**) Representative images and statistical analysis of cell migration assay in control and TIMP1-deficient endothelial cells at the indicated times. (**D**) Representative images and statistical analysis of EdU assay in control and TIMP1-deficient LN229 cells. *, P< 0.05; **, P< 0.01; ***, P< 0.001; **** P< 0.0001.

To fully determine the effect of TIMP1 on glioma cells, we knocked down TIMP1 in LN229 cells by transfecting specific siRNA (Figure 10A, 10B). Knockdown TIMP1 significantly inhibited the migration of LN229 cells (Figure 10C). In addition, EdU assay showed that TIMP1 could significantly affect the proliferation of LN229 cells (Figure 10D).

## DISCUSSION

Glioma is considered to be one of the most devastating tumors in adults [21]. Currently, maximal safe surgical resection followed by radiotherapy with concurrent temozolomide chemotherapy is still the standard treatment of GBM [22]. However, the median survival of GBM is poor, not exceeding 16 months [5]. In the present study, we built a survival nomogram prediction model, incorporating grade, age, IDH status, and risk score into the model to improve the prediction accuracy of the model.

Inflammation in the tumor microenvironment is an important manifestation of malignant tumors. Chronic inflammation has been proved to be closely related with carcinogenesis. The inflammation-enriched tumor microenvironment has been shown to be responsible for the progression of developing tumors into highly malignant neoplasms, including GBM. Macrophages, as the main inflammatory cells in the tumor microenvironment, regulate the activity of signaling pathways together with glioma cells, and ultimately promote cancer progression, tumor cell migration and invasion, and immunosuppression [23]. Here, we revealed the relationship between TAM and malignancy of glioma, demonstrated the value of TAM related signature in predicting the prognosis of glioma.

Based on superior computational methods and multiple cohorts, we screened core genes and constructed prediction models. The results provided evidence for TAMs-related signature as a valuable prognostic biomarker in glioma. Nine positive prognostic genes (MYL12A, MSN, S100A4, CHI3L1, PLAUR, EMP3, CASP4, TIMP1 and CCDC109B) were included in the prediction model. CHI3L1 is an abundant glycopolymer, which is synthesized and secreted by macrophages and various cells. CHI3L1 has been shown to be associated in multiple cancers [24]. Lately, Chen et al. showed that CHI3L1 promoted macrophage-mediated immune suppression by forming complexes with galectin 3 or galectin 3-binding protein [25]. As a member of the ERM family, MSN localized to filopodia and other membranous protrusions that connects the actin-based cytoskeleton to plasma membranes [26]. In glioblastoma, MSN could increase CD44 expression driven by the Wnt/β-catenin signaling pathway [27]. MYL12A was proved to be involved in DNA damage repair and p53-driven apoptosis [28]. TIMP1 is member of natural inhibitors of the matrix metalloproteinases, which has the effect of controlling the polarization of Natural Killer cells induced by the tumor-related cytokine TGFβ [29]. CCDC109B was involved in the activation of cell death pathway. Xu et al. showed that it plays an important role in mediating the migration and invasion of glioma cells induced by HIF1α [29].

As mentioned above, in previous studies, the role of some prognostic genes in glioma or other cancers have been reported. But we uncovered their potential role on tumor-infiltrating immune cells, which together with cancer cells constitute the tumor microenvironment. Due to the limitations of the research on the signaling pathways involved in target prognostic genes and their realistic role in tumor-associated macrophage infiltration, further researches are needed to explore the molecular mechanism.

## CONCLUSIONS

Overall, we revealed the relationship between TAM and malignancy of glioma, demonstrated the value of TAM related signature in predicting the prognosis of glioma, and provided potential targeted therapy for glioma.

## Supplementary Material

Supplementary Figures
