# Supplementary material for: Tumor-associated macrophages related signature in glioma
Source: Aging (Albany NY). 2022 Mar 24;14(6):2720–35. doi: 10.18632/aging.203968 (PMC9004565; doi:10.18632/aging.203968)
Supplement: Supplementary Figures [file aging-14-203968-s001.pdf]

SUPPLEMENTARY FIGURES

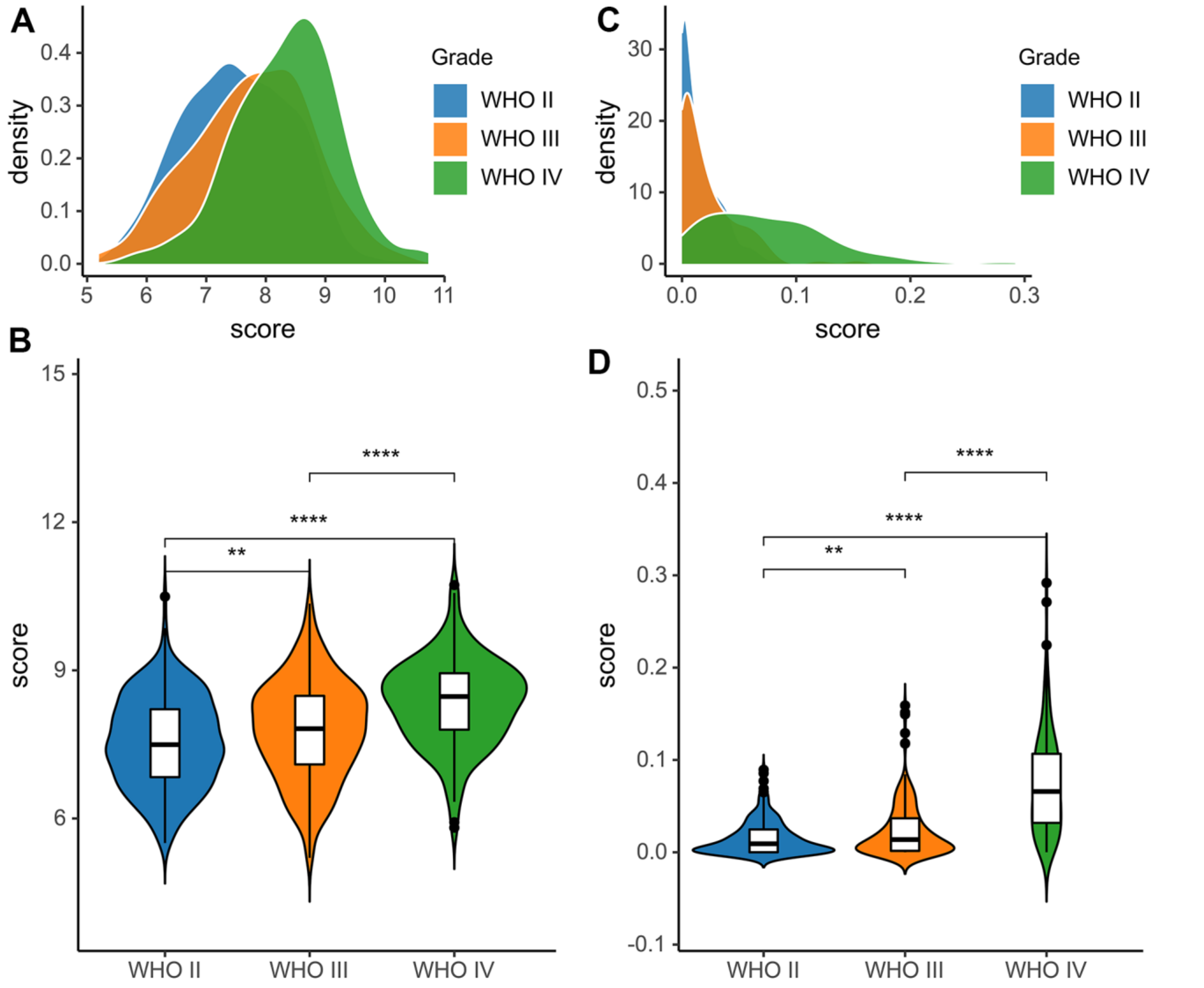

Supplementary Figure 1. Analysis of TAM in the TCGA dataset. (**A**, **B**) The TAM was analyzed by MCPCOUNTER method. (**C**, **D**). The TAM was analyzed by XCELL method.

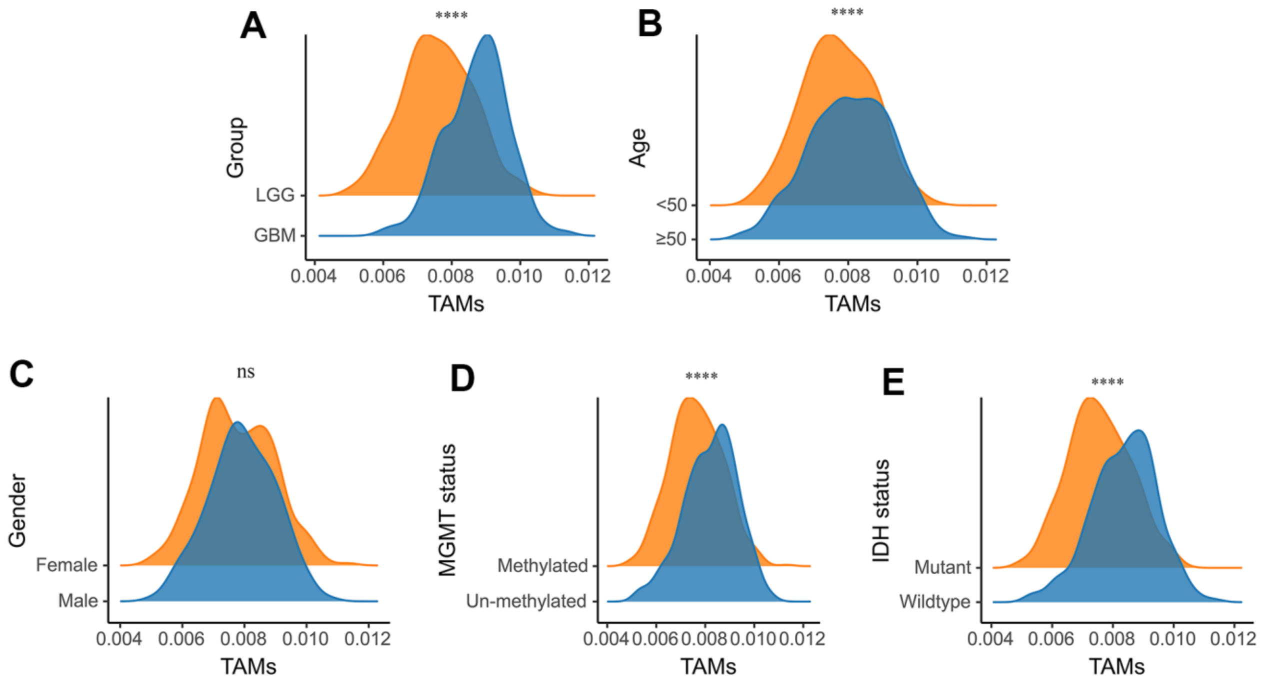

Supplementary Figure 2. Analysis of TAM in different WHO grade (**A**), age (**B**), gender (**C**), MGMT status (**D**) and IDH mutation status (**E**).

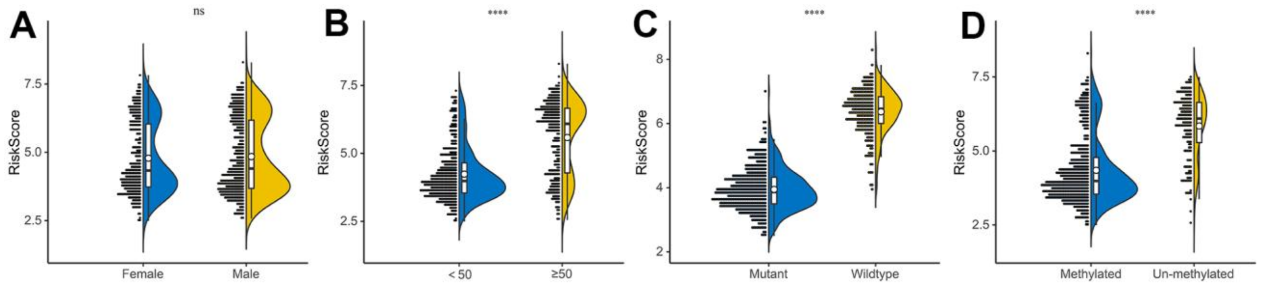

Supplementary Figure 3. The relationship between risk score and different gender (**A**), age (**B**), IDH mutation status (**C**), and MGMT status (**D**).
